# Case of Placenta Prævia

**Published:** 1850-02

**Authors:** W. F. Vidal

**Affiliations:** Aveley, Essex, late House Surgeon to the London Hospital


### OBSTETRICS.

*Case of Placenta Prævia.* By W. F. Vidal, M. R. C. S., Aveley, Essex, late House Surgeon to the London Hospital.—Emma T., aged 37, wife of a waterman, a woman of a full habit, being pregnant for the ninth time, wished to be attended by me in the event of her sending, as she felt very unwell, and at the same time stated, that the previous morning, and also three times before, she had a profuse discharge of blood, the first time being about the third month of utero-gestation, which gradually ceased on lying down. I recommended quietude, the recumbent position as much as possible; there being no hæmorrhage at that time, objected to an examination per vaginam, and as she was at the full period of her time, desired, that if a similar attack returned, to send without delay.

I heard no more of the case until five days after, when I was summoned at about half-past five, A. M., to go immediately to my patient, as she was in a very low state. I promptly attended, as, from the previous losses of blood, my suspicion was a case of placental presentation. On entering the room, I found her in the semi-erect posture in the bed, with wet napkins applied to the pubis, and in a very low state; countenance pallid, pulse rapid and extremely feeble; the bed, &c., was saturated with blood. On inquiry, I learnt that she had an attack similar to the first the same day after she had spoken to me, and also symptoms of approaching labor, both of which continued in a slight degree for three days, when the discharge ceased, the pains remaining, but less severe. On the following day, she exerted herself all day in her household duties, and felt great heaviness for sleep during the day, which increased at night; she went to bed, and, after a short time, all pains left her and she fell into a sound sleep and continued to do so until five o'clock the following morning, when she awoke with a feeling of faintness and general debility, and lying in a pool of blood, which grearly alarmed her, and immediately I was sent for. After some trouble I got her to the foot of the bed, as she dreaded to move from her position, and, on an examination per vaginam, found the thick mass of the placenta situated over the os uteri, and almost completely detached, with the exception of a small portion near the left side of the womb. The mouth of the latter dilated to an extent sufficient to allow nearly the introduction of the hand, and inclining to yield. During my examination a gush of blood took place, which immediately induced me to separate the remaining adherent portion of the after-birth, and endeavored to find the feet of the child, which I soon managed, and, in the act of turning, a sudden return of hæmorrhage occurred, which drove the placenta, together with some clots of blood, by my arm externally ; I ligatured the cord by the assistance of the nurse, deeming it might be of some object to the infant, although, from all appearances, " dead," as there was no pulsation in the cord, and a quantity of meconium passed ; and also what might be expected from the small quantity of nutrition received, if one may judge from the proximity of the placenta, in this instance, being so slight to the uterus.

As soon as the operation of turning had been accomplished the woman seemed so exhausted that I thought further proceedings would have been attended with great hazard, and therefore waited for a time until she rallied, at the same time keeping my hand in the mouth of the uterus as a plug, and I gave slight stimuli ; after a short time she regained her strength, pulse rose, and she expressed herself better. My wish was then to empty the uterus as soon as possible, which was done with comparatively little difficulty ; after which, to my surprise, " there being no hæmorrhage at the time," she became perfectly insensible, and remained in that state for some minutes, when consciousness gradually returned, but not the power of articulation ; the pulse extremely low. I ordered an egg beaten up with brandy, which seemed to have the effect of restoring, in part, the vital functions. The uterus contracted steadily and firmly, and no return of the flooding, which I attribute greatly to the syncope after the delivery. I remained some time with her, and left her as comfortable as possible after so severe a trial, and gave an anodyne mixture of tincture of opium and camphor mixture to be taken every three hours, and on visiting her in the evening, was pleased to find that she was perfectly composed, pulse tranquil, slight fever, and she had slept. My patient did well, and is now in perfect health.

My success in this case I impute to prompt and active measures, which, in one so severe, is, in my opinion, the sole hope of a favorable termination. At the same time, a constitution so good as that of my patient tends much to ultimate recovery.—*Lancet.*

